# Belladonna in the Nocturnal Incontinence of Urine in Children

**Published:** 1849-07

**Authors:** 


### ARTICLE V.

*Belladonna in the Nocturnal Incontinence of Urine in Children.*

"M. Trousseau narrates the case of a girl, five years old, who since her third year had been a victim of this obstinate complaint. No effort was neglected on the part of the parents to remove the habit; but all the means adopted, some of them sufficiently severe, were without effect. A pill containing one centigramme of the powder and half a centigramme of the extract of belladonna, was ordered to be taken every night at bed-time. During the first week two nights were passed without accidents; and from that time, with two or three exceptions, the complaint entirely disappeared. The treatment was resumed from time to time for nearly a year. This is only one of several cases occurring as well in his own practice as in that of M. Bretonneau, in which Prof. Trousseau has observed marked benefit from the use of this drug." *L'Union Med.*

"In a more recent number (Oct. 21) of the same Journal, Dr. Blache, physician to the Hopital des Enfans, records two very obstinate cases of nocturnal incontinance of urine occurring in individuals, one fifteen and the other eighteen years of age, where mercurial and sulphurous baths, refrigerant and astringent applications, tonic and ferruginous medicines, tannin, ergot of rye. nux vomica, and all other means had failed. Ultimately belladonna was exhibited with complete success.

